# Immune Regulation of Heme Oxygenase-1 in Allergic Airway Inflammation

**DOI:** 10.3390/antiox11030465

**Published:** 2022-02-26

**Authors:** Zhenwei Xia, Wenwei Zhong

**Affiliations:** 1 Department of Pediatrics, Ruijin Hospital Affiliated to Shanghai Jiao Tong University School of Medicine, 197 Ruijin 2nd Road, Shanghai 200025, China; 2 Department of Pediatrics, Xinhua Hospital Affiliated to Shanghai Jiao Tong University School of Medicine, 1665 Kongjiang Road, Shanghai 200090, China

**Keywords:** heme oxygenase-1, asthma, airway inflammation, immunoregulation

## Abstract

Heme oxygenase-1 (HO-1) is not only a rate-limiting enzyme in heme metabolism but is also regarded as a protective protein with an immunoregulation role in asthmatic airway inflammation. HO-1 exerts an anti-inflammation role in different stages of airway inflammation via regulating various immune cells, such as dendritic cells, mast cells, basophils, T cells, and macrophages. In addition, the immunoregulation role of HO-1 may differ according to subcellular locations.

## 1. Introduction

The primary physiological function of heme oxygenase (HO), a rate-limiting enzyme in heme metabolism, is to degrade heme into biliverdin (BV), free ferrous ion (Fe^2+^), and carbon monoxide (CO). BV is converted to bilirubin (BR), and iron is sequestered into ferritin. HO has two main isoforms, HO-1 and HO-2, of which HO-1 is an inducible isoform with molecular weight 32-kD. HO-1 is considered a protective protein. HO-1 is encoded by HMOX1 and regulated transcriptionally. HO-1 is expressed at low levels or is absent in most tissues except the spleen and liver under homeostatic conditions and highly expressed in response to various stimuli related to cellular stress and pro-oxidant signals, such as reactive oxygen species (ROS), cytokines, inflammatory mediators, and infection. The upregulation of HO-1 or its enzymatic products CO, BV, and BR have been shown to have anti-inflammatory, antioxidant, cell cycle regulation properties in vitro study or animal models of a variety of diseases including asthma [1,2,3].

Asthma is a chronic inflammatory disease with complex pathogenesis. Different triggers could lead to different types of inflammation. For example, exposure to an allergen in sensitized individuals tends to invoke Th2 high airway inflammation characteristic of increased Th2 cytokine and eosinophil (EOS) infiltration in the airway. On the other hand, respiratory infection or recurrent environmental tobacco smoke exposure tend to induce Th17 or Th1-dominant immune responses and Th2-low airway inflammation characteristic of neutrophil infiltration. A variety of immune cells are involved despite Th2-high or low airway inlfammation. HO-1 expression is significantly upregulated in many immune cells such as dendritic cells (DCs), macrophages [4], mast cells (MCs) [5], basophils (BAs) [6], and T cells [7] in response to cellular stress. Both HO-1 and CO displayed anti-inflammatory, antioxidative stress, and immune regulation properties in an asthma animal model [8,9] and inhibited the proliferation of cultured human airway smooth muscle cells (HASMCs) through downregulation of ERK1/2 activation, which indicated the anti-airway remodeling effects of HO-1. HO-1 has been shown to exert anti-inflammatory effects in both T helper cell type (Th) 2-dominant [6,10,11] and Th17-dominant models of asthmatic airway inflammation [12]. In this article, we will discuss mechanisms by which HO-1 regulates immune responses during allergic airway inflammation with a focus on specific immune cells in different stages of inflammation.

## 2. Mechanisms of Asthmatic Airway Inflammation and Role of HO-1

Asthma is a heterogeneous disease characterized by chronic airway inflammation and bronchial hyperresponsiveness. As a protective protein, many studies prove that HO-1 expression is upregulated both in asthma patients [13,14,15] and animal models of asthma [6,16,17,18]. The pathophysiological significance of this phenomenon is to avoid further deterioration of inflammation since inhibition of endogenous HO-1 further aggravates the inflammation [9,10]. Furthermore, upregulation of HO-1 or exogenous administration of CO and bilirubin which is produced by its degradation of heme has significant protective effects on allergic airway inflammation. These factors can inhibit plasma exudation to the trachea, main bronchi, and segmental bronchi; reduce infiltration of inflammatory cells (such as EOS, neutrophils, lymphocytes, and macrophages) around the airway and in bronchoalveolar lavage fluid (BALF); alleviate airway reactivity and mucus secretion [19,20,21,22]; decrease the proportion of antigen-specific Th2 cells [6] and Th17 cells [12,23] in mediastinal lymph nodes and the spleen; and further inhibit allergic airway inflammation. These findings suggest that HO-1 has a protective effect in various types of asthmatic airway inflammation.

The pathogenesis of asthma is complex, and immune imbalance is the most important mechanism of asthma. Th2 immune response-dominant asthma (the most common phenotype) involves EOS inflammation, while Th17 immune response-dominant asthma (the primary refractory phenotype) involves neutrophil inflammation. Regardless of type, this immune disorder proceeds through initiation, Th cell-directed differentiation, amplification, and effective stages. The initiation stage is particularly important because it determines the direction of the immune response. During the initiation of immune responses, antigen-presenting cells (APCs) and T cells interact with each other via the T cell receptor (TCR)–major histocompatibility complex (MHC) peptide complex and costimulatory molecules on the cell surface. In the context of specific cytokine environments, these interactions direct naïve T cell differentiation into antigen-specific Th cells (Th1, Th2, and Th17). Thus, the state and microenvironment of APCs determine the type of T cell immune response. During the effective stage, cytokines secreted by Th2 cells infiltrate around the airway along with EOS, MCs, and BAs, resulting in further release of inflammatory mediators that promote EOS infiltration-domain allergic airway inflammation. Th17 cells, mainly chemotax neutrophils, cause neutrophil-dominant airway inflammation [24,25,26,27]. Thus, various immune cells participate in the formation of chronic airway inflammation during asthma onset. HO-1 has important regulatory effects on multiple types of immune cells involved in airway inflammation [6,12,19,20,21,22,23].

## 3. HO-1 Inhibits Inflammation during the Initial Stage

### 3.1. Inhibition of DC Function

APCs play a key role in the initialization of adaptive immunity via promoting T cell differentiation. APCs capture antigens from the external environment via endocytosis or phagocytosis and degrade them into peptide fragments and binding with MHC II molecules. Antigen peptide–MHCII complex-loaded APCs then contact with naïve T cells via TCRs on the surface and initiate T cell polarization. DCs are the most important professional APCs [28,29]. This function of APCs is regulated by pathogen-associated molecular patterns (PAMPs) via pattern recognition receptors (PRR) [28,30,31]. HO-1 is constitutively expressed in immature DCs (iDCs) such as human monocyte-derived iDCs, freshly isolated rat splenic DC subsets, and rat bone marrow-derived iDCs, and is downregulated during DC maturation [32]. Signaling through PAMPs and its receptor can also regulate HO-1 expression in APCs [28,32,33]. In a mouse model of allergic airway inflammation, reinfusion of DCs highly expressing HO-1 significantly alleviated allergic airway inflammation [34], suggesting a regulatory effect of HO-1 on the antigen-presentation function of DCs. HO-1 can regulate DCs through multiple modes, such as effects on maturation, antigen presentation, and release of cytokines and extracellular vesicles (EVs). EVs are membranous structures loaded with various proteins, lipids, and nucleic acids and play important role in cell–cell communication.

First, HO-1 can inhibit DC maturation. Inhibition of HO-1 in DCs promotes the maturation of DCs [32,35,36], whereas over-expression of HO-1 was shown to inhibit maturation of bone marrow-derived DCs presenting a tolerance phenotype, as well as the presentation of exogenous soluble antigen to naïve T cells [34,37,38,39,40,41], which further affect the polarization of Naïve T cells towards Th1, Th2, and Th17 cells subsets. The function of APCs is regulated by PAMPs, HO-1 and its end-product CO can inhibit DC maturation by interfering with PAMPs and receptor binding. For example, CO can modify the natural conformation of toll-like receptor 4 to reduce DC maturation [42,43]. Thus, similar impairments of key steps required for correct conformational assembly of this complex on the surface of APCs are likely to reduce DC sensitivity to LPS stimulation by interfering with LPS recognition. In addition, upregulation of HO-1 activity renders DCs insensitive to LPS-induced activation of the p38 mitogen-activated protein kinase/cAMP-response element-binding protein/activating transcription factor 1 signaling pathway [38]. Importantly, all of the above factors could influence the LPS-induced maturation of DCs through effects on APCs.

Second, HO-1 and CO can inhibit antigen presentation to regulate DCs. In the process of antigen presentation, APCs first capture antigen components and endocytose them to form early endosomes, late endosomes, and fuse with proteasome/MHC molecules containing endosomes; then, they can fuse with lysosomes to form an MHC peptide complex. HO-1 and CO not only reduce the capability of APCs to identify PAMPs but also impair fusion between late endosomes and lysosomes [40], reduce mitochondrial membrane potential and ATP production in DCs, impairing cargo transport and endosome-to-lysosome fusion [39]. Disrupting fusion between antigen-containing late endosomes and lysosomes further blocks antigen transport by preventing the formation of MHC-II-peptide fragments in lysosomes, thus inhibiting the presentation of soluble antigens by DCs.

Third, HO-1 can regulate patterns of cytokines released by DCs. DCs highly expressing HO-1 secrete high levels of interleukin 10 (IL-10) and TGF-β, and low levels of IL-12 and IL-23, yielding a microenvironment conducive to the differentiation of naïve T cells into regulatory T cells (Tregs) rather than Th2 or Th17 cells [32,34]. In addition, overexpression of HO-1 in DCs can inhibit DCs maturation as we discussed above and direct naïve T cells polarization towards Treg subtypes [34]. The absence of HO-1 in APCs abolished the suppressive activity of Treg cells on effector T cells, indicating that HO-1 activity in APCs is important for the inhibitory function of Tregs [44]. This evidence indicates that the regulatory role of HO-1 on Tregs partly via APCs inhibitory manner.

Finally, HO-1 can regulate immune responses by inhibiting the release of EVs from DCs. DC-derived EVs lead to allergic airway inflammation by presenting allergens and directly contacting CD4^+^ T cells. Our previous study found that stimulating DCs with dust mite extract expressing MHC II resulted in the concentrated release of EVs, which induced Th2 cell differentiation in vitro. In an animal model of asthma, concentrated EVs were produced following house dust mite stimulation of the airway, indicating typical allergic airway inflammation. In hemin-induced EV-sensitized mice, allergic airway inflammation was significantly alleviated; EOS infiltration and mucus secretion were reduced in the airway; levels of IL-4, IL-5, and IL-13 were decreased in the lung; numbers of Th2 cells in the mediastinum lymph node (MLN) were decreased; numbers of Treg cells in MLN were increased; and numbers of Th17 cells were reduced. These results suggest that the anti-inflammatory effects of EVs are executed through regulation of Th17/Treg balance and inhibition of Th2 and Th17 cell proliferation [45].

### 3.2. Inhibition of BA Function

In addition to DCs, BAs are an important APC for initiating allergic inflammation. Although DCs have historically been considered an important APC for initiating T cell immune responses and forming memory immune cells, they cannot secrete IL-4 and independently initiate Th2 immune response. Recently, the role of BAs in Th2 immune responses and allergic diseases has attracted increased attention. We and others have confirmed that BAs with antigen-presentation functions express costimulatory molecules and secrete “early IL-4”. Moreover, BAs can promote Th2 cell differentiation without exogenous IL-4 in vitro [46,47,48,49]. Currently, BAs are considered to both assist APCs (such as DCs) in the initiation of directional differentiation of Th2 cells by secreting Th2 cytokines (such as IL-4) and independently initiate Th2 immune responses as APCs [50]. BAs can also obtain MHC II–peptide complexes from DCs through trogocytosis to exert APC function [47]. Furthermore, our previous study demonstrated HO-1 expression in BAs by immunohistochemistry. Overexpression of HO-1 significantly inhibited the expression of activation marker CD200R and costimulatory factors, inhibited IL-4 release stimulated by DNP-OVA/anti-DNP-IgE, inhibited DQ-OVA up-taken both in the lung-derived BAs from asthma animal models and in cultured bone marrow-derived BAs, and subsequently, inhibited polarization of naïve T cells into Th2 cells in vitro and inhibited OVA-induced allergic airway inflammation and the Th2 immune response.

## 4. HO-1 Inhibits Inflammation during the Effective Stage

### 4.1. HO-1 Promotes Treg Cell Function and Inhibits Th2- and Th17 Cell-Mediated Inflammation

The imbalance of the Th cell subgroup plays an important role in the pathogenesis of asthma. HO-1 inhibits Th cell functions via different mechanisms. Firstly, CO, which is one of the end-products of HO-1, can inhibit the proliferation of CD4^+^ T cells by blocking TCR-dependent IL-2 production [51]. Another end-product, BR, can inhibit CD4^+^ T cells by inducing apoptosis, suppressing co-stimulatory molecule expression in CD4^+^ T cells, and inhibiting CD4^+^ cell proliferation [52].

Secondly, HO-1 can regulate the balance of the Th cell subgroup via Tregs. Tregs are important immune cells to maintain immune homeostasis. Tregs inhibit effector T cells proliferation and function via interactions with negative costimulatory molecules, secrete suppressive cytokines IL-10, and competition for IL-2 [53], and subsequently exert inhibitory effects on Th1, Th2, and Th17 cell-mediated inflammation [54,55,56]. HO-1 promotes Tregs function, which is regarded as an important mechanism for its immunomodulatory function. HO-1 expression is significantly different between CD4^+^ CD25^+^ Treg cells and CD4^+^ CD25^+^ T lymphocytes [7], and is consistent with Foxp3 expression in these two cell types. Transfection of Foxp3 into Jurkat T cells significantly upregulated the expression of HO-1 and inhibited their proliferation and cytokine production in a cell contact-dependent manner. Treatment of freshly isolated CD4^+^ CD25^high^ from the spleen with hemin or transfected with an HO-1 expression vector (pcDNA3HO-1) in vitro not only significantly enhanced Foxp3 expression and IL-10 secretion but also enhanced its ability to inhibit effector T cell proliferation. The regulatory role of HO-1 was significantly inhibited by the addition of an HO-1 activity inhibitor [11,57]. In an animal model of asthmatic allergic airway inflammation, overexpression of HO-1 induced by hemin enhanced proportions and functions of CD4^+^ CD25^+^ Treg cells [10,11] and alleviated OVA-induced allergic airway inflammation. On the contrary, inhibition of HO-1 activity with tin-protoporphyrin reversed the above effects of HO-1 [10,11]. These in vivo and in vitro studies show that HO-1 plays an important role in regulating Treg function, However, the direct role of HO-1 in regulating Treg function is challenged since HO-1-deficient mice not only exhibited a significantly higher proportion of Foxp3-expressing cells among total CD4^+^ and CD4^+^ CD25^+^ cells in comparison to wild type mice but also displayed a similar inhibitory role in suppressing the proliferation of effector T cells in vitro. In the same study, HO-1-deficient APCs abolished the suppressive activity of Treg cells [44], indicating that HO-1 may regulate CD4^+^ CD25^+^ Treg cells by indirectly promoting Treg differentiation through inhibition of DC maturation. Considering Tregs have inhibitory effects on T cell subsets of Th1, Th2, and Th17 cells, we speculate that HO-1 enhances Treg function and therefore regulates the balance of Th1, Th2, and Th17 cells.

### 4.2. HO-1 Inhibits Th17 Cell-Mediated Inflammation

Th17 cells, an important T cell subset in asthma, play key roles in refractory asthma and neutrophil-dominant asthma types by promoting neutrophil growth, development, and chemotactic aggregation in the airway. Th17 cells achieve these effects by secreting cytokines and are essential for inducing neutrophil infiltration-dominant asthma [26,58,59]. Overexpression of HO-1 inhibited the differentiation of naïve T cells into Th17 cells, as well as the secretion of IL-17A in vitro [60]. In an animal model of non-eosinophilic asthma, upregulation of HO-1 expression significantly reduced the proportion of Th17 cells, promoted IL-10 expression, reconstructed the balance of Th17/Treg cells in vivo, and subsequently inhibited Th17 cell-mediated neutrophilic airway inflammation. In contrast, inhibition of HO-1 activity reversed the inhibitory effect of HO-1 on neutrophil airway inflammation and activation of the Th17 cell signaling pathway [12].

### 4.3. HO-1 Inhibits MC Function

MCs are important effector cells in asthma. Sensitized MCs can be activated and degranulated to release various preformed mediators and pre-synthesized mediators, such as proteases, cytokines, chemokines, and arachidonic acid metabolites. MCs can de novo synthesize lipid mediators by enzymes located in the plasma membrane and synthesize mRNAs encoding cytokines and chemokines. MCs can also regulate inflammation via the secretion of exosomes containing regulatory molecules [61,62,63]. MCs participate in asthmatic inflammation which is characterized by inflammatory cell infiltration, microvascular leakage, airway hyperresponsiveness, bronchoconstriction by degranulation, and the secretion of various mediators [64,65,66]. Moreover, MCs participate in allergic inflammation via regulating T cells, DCs, and other inflammatory cells, which results in further chemotaxis, infiltration, and activation of EOS, neutrophils, and other inflammatory cells in the airway [61,67]. Recent studies revealed that MCs can regulate Th17-mediated autoimmune diseases by inducing Tregs [64].

HO-1 has important regulatory effects on MC function. HO-1 is expressed in MC [5] and MC cell lines [68], and can be induced during MCs degranulation [5,68] and upregulation of HO-1 decreased MCs degranulation induced by complex 48/80 and leukocyte adhesion to blood vessels [5]. Upregulation of HO-1 during MCs degranulation was related to cellular oxidative stress since upregulation of HO-1 was inhibited by antioxidant N-acetyl-L-cysteine [68]. HO-1 and its end-products, BR and BV, can inhibit adhesion and degranulation of MCs [68]. HO-1 can also inhibit the production of inflammatory mediators in MCs by selectively inhibiting the DNA-binding activity of the AP-1 transcription factor [69].

In addition, HO-1 regulates MC-mediated immune regulation. Co-culture of MCs and DCs led to a significant release of tumor necrosis factor-α (TNF-α), IL-6, and interferon (IFN), which promoted DC maturation. Upregulation of HO-1 in MCs before co-culture with DCs inhibited expression of costimulatory molecules on DCs and inhibited DCs maturation [70]. In contrast, downregulation of HO-1 expression promoted MC degranulation, DC costimulatory molecule expression, and DC maturation. These findings suggest that upregulation of HO-1 in MCs stabilizes the MC membrane and prevents its degranulation, thereby maintaining the DCs in an immature state to ultimately alleviate the immune response [39].

### 4.4. HO-1 Regulated Inflammation by Inhibiting NLRP3 Inflammasomes

Factors such as environmental irritants and respiratory infections are common triggers of asthma exacerbation and neutrophilic airway inflammation responds to these situations. Signaling through inflammasome activation plays a key role in neutrophilic airway inflammation. The inflammasome is an intracellular protein complex and activated by ligation of PAMPs and its receptor. Upon ligand sensing, inflammasome components assemble and self-oligomerize, followed by autoactivation of caspase-1 and leading to cleave pro-IL-1β and pro-IL-18 to IL-1β and IL-18, T helper 17 activation, IL-8/IL-6 overproduction, thus initiating or aggravating neutrophilic airway inflammation. Inflammasomes also involved in caspase-1-mediated pyroptosis [71,72]. Among the known inflammasomes, nucleotide-binding domain and leucine-rich repeat protein 3 (NLRP3) are crucially involved in the pathogenesis of asthmatic airway inflammation [73].

HO-1/CO has a potential regulatory role in inflammasome signaling. Li and colleges [74] demonstrate that the induction of HO-1 by hemin inhibited LPS-induced production of IL-1β, inhibited NLRP3 inflammasome activation in human gingival epithelial cells in vivo. Luo and colleges [75] also demonstrated that upregulation of HO-1 by hemin inhibited LPS-induced NLRP3 inflammasome activation, reducing IL-1β and IL-18 production in sepsis-induced acute lung injury. On the contrary, inhibition of HO-1 activity reversed the above results. On the other hand, CO can inhibit LPS and ATP-induced caspase-1 activation and production of IL-1β and IL-18 in bone marrow-derived macrophages. Treatment of CO-releasing molecule-2 (CORM-2) both inhibited NLRP3 inflammasome activation in LPS-induced acute lung injury and ER stress-induced inflammation [76]. CORM2 also can inhibit caspase-1activiation [77,78,79] and thioredoxin-interacting protein (TXNIP)–NLRP3 complex formation [77,78]. Our previous study showed that HO-1 products, CO and BR, inhibited the NLRP3–RXR axis and NLRP3 inflammasome-mediated apoptosis of AECs, which inhibits subsequent production of IL-25, IL-33, thymic stromal lymphopoietin, and other pro-Th2 epithelial-derived cytokines. In addition, we found that HO-1 bound to the NACHT domain of NLRP3 and RXRα/RXRβ subunits, suggesting that the non-enzymatic action of HO-1 may be involved in the regulation of NLRP3 inflammasomes [80].

### 4.5. HO-1 Promotes Polarization of Macrophages to M2 Phenotype

Macrophages are one of the important inflammatory cells involved in the pathogenesis of asthma [81]. Macrophages are divided into M1 and M2 subpopulations according to their responses to environmental or inflammatory stimuli. M1 macrophages respond to pro-inflammatory cytokines, such as IFN-γ and TNF-α, and promote a local Th1 environment. M2 macrophages (alternatively referred to as activated macrophages) respond to IL-4 and IL-13, promote the production of the anti-inflammatory cytokine IL-10, and regulate Th2 immune responses [82,83,84]. HO-1 is considered a regulator of immune responses because it can promote the polarization of M2 macrophages. Indeed, HO-1 is highly expressed in M2 macrophage subsets, and its elevation in response to multiple stimuli can drive phenotypic transfer to M2 macrophages [85,86,87]. HO-1-knockout bone marrow macrophages (mHO-1-KO) exposed to LPS (M1-inducer) or IL-4 (M2-inducer) exhibited an enhanced M1 phenotype and inhibited M2 phenotype. In contrast, promotion of the M2 phenotype was observed in HO-1-overexpressing (HO-1-Tg) mice [88]. Collectively, these studies support the hypothesis that HO-1 promotes the M2 phenotype. However, there is a lack of studies demonstrating the role of HO-1 on the regulation balance between M1 and M2 subpopulations in clinical studies or animal models of asthma, and further studies are required.

## 5. Subcellular Localization and Anti-Inflammatory Mechanism of HO-1

It is well accepted for a long time that the biological functions of HO-1 are related to its enzymatic products CO, BR/BV, and ferritin. A recent study revealed that HO-1 exerts its function via interaction between other cellular proteins in an activity-independent manner, which is referred as “non-canonical effects of HO-1” in published studies. Those different fashions may in part relate to specific subcellular locations of HO-1.

HO-1 was initially identified in the endoplasmic reticulum (ER). HO-1 is mainly located in the ER under physiological conditions. Recently, more specific subcellular locations of HO-1 have been revealed. HO-1 was identified in mitochondria, plasma membrane, the caveolae, and the nucleus after various stimuli (e.g., LPS, or hypoxia) [89]. Varying the subcellular localization of HO-1 protein has various effects on its cell-protective functions which may depend on the structural integrity of the HO-1 protein. Except for the nucleus, HO-1 is fixed on the membrane by a transmembrane sequence (TMS) located at the carboxyl terminal of its protein structure, and the rest of the protein structure faces the cytoplasm and colocalizes with cytochrome P450 reductase (CPR) and BVR to facilitate heme degradation [90]. Cleavage of the TMS enables HO-1 relocation. On the other hand, CPR can stabilize the HO-1 protein structure to prevent its relocation and maintain its enzymatic activity by promoting oligomerization [91]. HO-1 locates in the mitochondria, vacuole, and plasma membrane and maintains full protein structure and enzymatic activity and coexists with biliverdin reductase [92,93], indicating that its function is achieved mainly through enzymatic activity. The functional significance of HO-1 in different cellular compartments remains unclear. In caveolae, HO-1 activity is negatively modulated by caveolin-1 (CAV1). It could serve as a brake on HO-1 function and provide a possible approach for the active extracellular transfer of HO-1 [94]. In mitochondria, HO-1 induces increased ROS, which appears important for its regulation of mitochondrial heme content, and plays an important role in apoptosis [89,92].

Unlike HO-1 in the ER, mitochondria, and caveolae, nuclear HO-1(NHO-1) exists in a truncated form (28 kDa) in the COOH terminal with a lack of enzyme activity to degrade heme [89]. Therefore, nuclear HO-1 may exert its roles independent of enzyme activity [89,95]. HO-1 in the ER will be truncated and translocate to the nucleus under pathological conditions or external stimuli, leading to cellular stress [94]. NHO-1 has been reported as a regulator of nuclear transcription factor activities such as NF-κB, AP-1, and Nrf2 [96], the latter of which regulates the antioxidant response. By regulating gene expression levels in the nucleus, HO-1 provides resistance to redox stimulation that can protect cells from dysfunction or death, making it an important part of signaling involved in the cellular response to oxidative stress. As the DNA-binding motif of typical transcription factors is not detected in the HO-1 protein structure and direct recruitment of HO-1 to DNA has not been observed, nuclear HO-1 is unlikely to regulate gene expression by directly binding DNA. It is speculated that HO-1 acts as a transcriptional coregulatory protein that binds transcription factors or complexes to regulate their DNA-binding affinity, thus indirectly regulating transcription of key response genes [89].

Our previous study investigated the non-enzymatic anti-inflammatory effects of HO-1. We found that HO-1 inhibited Th17 cell differentiation mainly by inhibiting IL-6-induced STAT3 phosphorylation and therefore inhibited activation of the STAT3/RORγT signaling pathway. Co-IP results indicated that endogenous HO-1 directly bound STAT3 rather than Jak1, JAK2, or SOCS3, suggesting that HO-1 inhibited STAT3 phosphorylation by interacting with STAT3. This result is consistent with the finding of Elguero and colleagues. In their study, they demonstrated that HO-1 and STAT3 bind to each other and therefore inhibit the phosphorylation of STAT3 and subsequent nuclear translocation of pSTAT3 in prostate cancer cells [97]. Our study demonstrated that co-transfection of 293T cells with plasmids containing HO-1 and STAT3 domains revealed that HO-1 bound all regions of the STAT3 protein except the helical domains, especially the transcriptional activation region (AA 689–770). Further experiments confirmed that HO-1 directly bound STAT3, in particular, the transcriptional activation domain-containing Tyr705 in the STAT3 protein. However, whether HO-1 plays an indirect regulatory role through intermediate proteins (for example, whether HO-1 leads to increased dephosphorylation of STAT3 by promoting SHP-1 activation) or inhibits phosphorylation by directly binding STAT3 requires further study [98].

## 6. Potential Clinical Application of HO-1

Results from basic research demonstrated that HO-1 alleviated allergic airway inflammation by its anti-inflammatory, antioxidative stress, and immune regulation properties. Moreover, how to apply those results to clinic use has attracted the interest of researchers. Currently, research focused on the value of HO-1 in both diagnostic and therapeutic applications. In view of diagnosis, clinical studies revealed that exhaled CO elevated in asthma patients and the levels of exhaled CO were associated with the severity of asthma and disease exacerbation [99,100,101], indicating that exhaled CO has potential value in asthma management as a noninvasive tool. On the other hand, therapeutic applications of drugs targeting HO-1 activity and expression have attracted significant attention, including pharmacologic modulators of HO-1, gene therapy, and enzymatic byproducts of HO-1 such as CO and CO-releasing molecules (CORM) [3,102,103,104]. Despite the achievement of attempts in animal studies with promising results, pharmacologic use of HO-1 targeted drugs still faces great challenges, especially safety and efficacy.

## 7. Conclusions

**Figure 1:**
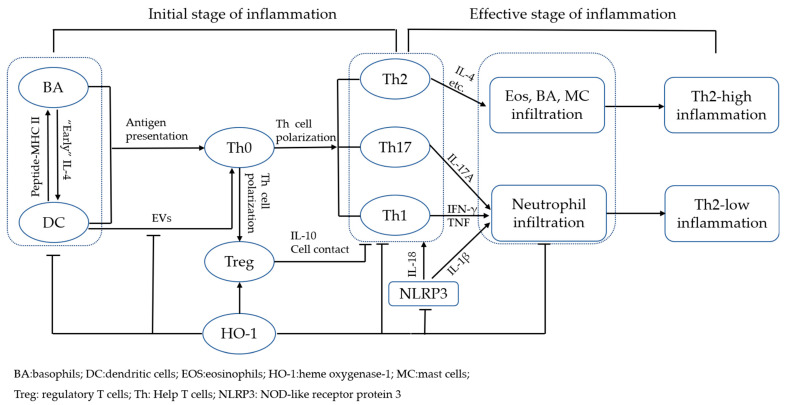
Multi-target effect of HO-1 and its immune regulation role in allergic airway inflammation. 1, HO-1 regulates APCs function and inhibits allergic airway inflammation at the initial stage: a, HO-1 and its end-product CO inhibit DC maturation by interfering with PAMPs and receptor binding, inhibit antigen presentation by impair fusion between late endosomes and lysosome, inhibit release of EVs from DCs and promote Treg polarization; b, BAs assist DCs participate in Th2 cells polarization by secreting “early” IL-4; BAs initiate Th2 polarization independently as APCs or BAs obtain MHC II-peptide complex from DCs through trogocytosis and subsequently initiate Th2 polarization; HO-1 inhibits BAs participate in Th2 cell differentiation by inhibits BAs activation, soluble antigen up-taken, expression of costimulatory molecules and secrete “early IL-4”; 2, HO-1 inhibits allergic airway inflammation at effective stage: a, HO-1 and its end-product inhibit CD4^+^ T cell proliferation and function directly or via promotion of Treg; b, HO-1 inhibits Th17 cell-mediated neutrophilic airway inflammation by inhibiting Th17 cell polarization and IL-17A releasing; c, HO-1 and its end-products suppresses mast cell degranulation and releasing of inflammatory mediators; d, HO-1 and its end-product inhibit NLRP3 inflammasome activation and subsequently inhibit IL-1β and IL-18 mediated airway inflammation.

**Figure 2:**
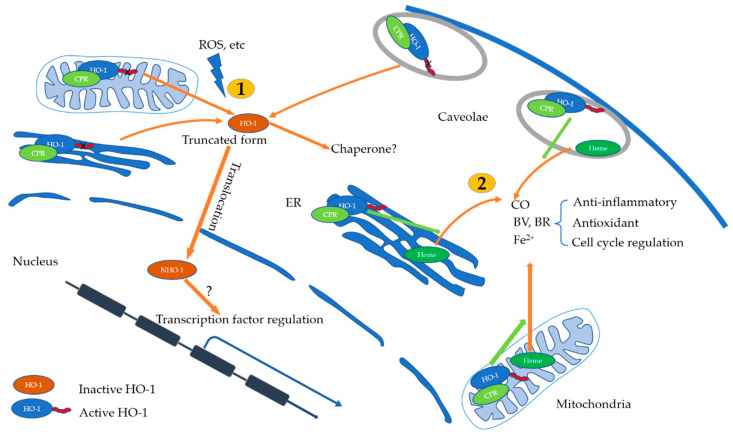
Subcellular localization and roles of HO-1. **1**. HO-1 locates in endoplasmic reticulum (ER), mitochondria, plasma membrane, and the caveolae. HO-1 is fixed on the membrane by a transmembrane sequence (TMS) and stabilized by cytochrome P450 reductase (CPR) to prevent relocation. It remains full protein structure and enzymatic activity and its function is achieved mainly through enzymatic activity. Its enzymatic products CO, BV, and BR have been shown to have anti-inflammatory, antioxidant, cell cycle regulation properties; **2**. HO-1 will be truncated and relocated under pathological conditions or external stimuli which leads to cellular stress. Truncated HO-1 lacks enzyme activity to degrade heme and may act as a chaperone to regulate the activity of signaling pathway proteins or as a transcription factor regulator.

During asthmatic airway inflammation, HO-1 regulates differentiation of Th1/Th2/Th17 cell subsets by inhibiting the functions of APCs (such as DCs and BAs), promotes Treg function, and suppresses allergic airway inflammation characterized by Th2-dominant EOS infiltration and neutrophil infiltration-dominant Th17 immune response. HO-1 directly inhibits airway inflammation by inhibiting BA, MCs, and certain functions during the inflammatory effective stage (summarized in Figure 1). HO-1 not only exerts anti-inflammatory and immunomodulatory effects through its enzymatic products CO and BR/BV but also regulates the transcription of key genes by acting as a transcription coregulatory protein with transcription factors or complexes in the nucleus (summarized in Figure 2). However, studies evaluating the non-enzymatic immunoregulatory mechanism of HO-1 are limited, which represents an important direction for future research.
